# Tailoring the Wettability of Colloidal Particles for Pickering Emulsions via Surface Modification and Roughness

**DOI:** 10.3389/fchem.2018.00225

**Published:** 2018-06-19

**Authors:** Meina Xiao, Anli Xu, Tongtong Zhang, Liangzhi Hong

**Affiliations:** Department of Polymer Materials Science and Engineering, South China University of Technology, Guangzhou, China

**Keywords:** Pickering emulsions, wettability alteration, interfaces, surface modification, surface roughness

## Abstract

Pickering emulsions are water or oil droplets that are stabilized by colloidal particles and have been intensely studied since the late 90s. The surfactant-free nature of these emulsions has little adverse effects such as irritancy and contamination of environment and typically exhibit enhanced stability compared to surfactant-stabilized emulsions. Therefore, they offer promising applications in cosmetics, food science, controlled release, and the manufacturing of microcapsules and porous materials. The wettability of the colloidal particles is the main parameter determining the formation and stability of Pickering emulsions. Tailoring the wettability by surface chemistry or surface roughness offers considerable scope for the design of a variety of hybrid nanoparticles that may serve as novel efficient Pickering emulsion stabilizers. In this review, we will discuss the recent advances in the development of surface modification of nanoparticles.

## Introduction

Emulsions stabilized by solid particles, so-called Pickering emulsions, offer promising applications in cosmetics, food science, controlled release, and the manufacturing of microcapsules and porous materials (Aveyard et al., 2003; Dickinson, 2010; Rayner et al., 2014; Silverstein, 2014; Marquis et al., 2016; Marto et al., 2016; Wu and Ma, 2016). The surfactant-free nature of Pickering emulsions has seldom adverse effects such as irritancy and contamination of environment and typically exhibit enhanced stability compared to surfactant-stabilized emulsions. Generally, a wide range of colloidal particles including polystyrene latexes, inorganic particles, microgels, organic pigment particles, and gelatin particles have been demonstrated to be effective Pickering emulsion stabilizers (Ashby and Binks, 2000; Binks and Lumsdon, 2001; Binks and Whitby, 2004; Li and Ngai, 2013; Binks and Olusanya, 2017; Tan et al., 2017).

The stability of Pickering emulsion is influenced by many factors, including particle size, shape and concentration, as well as surface wettability. Most research has concluded that the main parameter controlling the particle interfacial behaviors and the emulsion stability is the wettability of particles, which can be measured by the three phases contact angle at the oil-particle-water interface. The particles have to be partially wettable by both water and oil phases. If the particle is too hydrophilic, it will prefer to stay in aqueous phase rather than go to the interface. If the particle is too hydrophobic, it will prefer to stay in the oil phase. The energy required to remove a particle from an oil/water interface is given by the following equation:

$$\begin{array}{r} \Delta E=\;\pi r^{2}\gamma_{O/W}{\left(1-|\cos\theta_{w}|\right)}^{2} \end{array}$$

where *r* is the radius of particles, γ_*o*/*w*_ is the oil/water interfacial tension and θ_*w*_ is the three phase contact angle (Binks, 2002). For microparticles with intermediate contact angles, the detach energy is much greater than the thermal energy and the absorption of particles at the oil/water interface is irreversible. Empirically, the emulsion type is correlated to which phase the particle prefer to disperse. Preferentially water-wetted particles favor to stabilize oil-in-water (O/W) emulsions, and preferentially oil-wetted particles favor to stabilize water-in-oil (W/O) emulsions.

In this review, we highlight recent advances in tuning the wettability of colloidal particles for Pickering emulsions and related applications, especially focus on switchable Pickering emulsions, environmentally-responsive properties, and the effect of surface roughness. There have been several reviews published devoted to Pickering emulsions (Tang et al., 2015; Wang and Wang, 2016). Wang and coworkers provide a review of controllable Pickering emulsions by adjusting amphiphilicity of soft particles, rigid particles and Janus particles (Wang and Wang, 2016). Tang and coworkers provide a comprehensive review of stimuli-responsive Pickering emulsions (Tang et al., 2015). However, our goal in this short review is to provide detail discussion of surface modification methods of tuning the particle wettability by either surface chemistry or surface topology. In addition to tune the wettability by surface chemistry with small molecules or polymers, surface topology such as surface roughness also contributes to the wettability of particles. The typical functionalization methods of particles is summarized in Table 1 with short comments.

**Table 1:** Typical fabrication methods of tuning wettability of particles for Pickering emulsions.

**Method**	**Modifiers**	**Particles**	**Emulsion types and characteristics**	**References**
Physical adsorption	8-hydroxyquinoline	Silica	O/W; stable emulsions in a narrow pH range from 4.4 to 5.5	Haase et al., 2010
	Dialkyl adipate	Silica and Zirconia	O/W; the formation of Hydrogen bonds between oils and particle surfaces renders partially hydrophobicity	Binks and Yin, 2016
	Palmitic acid	Silica	W/O; simplicity, formation of nanoparticle-PA complexes at oil/water interfaces	Santini et al., 2014
	Oleic acid	Silica	O/W; long-term stability, biocompatible materials	Sadeghpour et al., 2013
	Surfactants and polymers	Clay	O/W; emulsions have either viscous or gel-like properties	Reger et al., 2012
	Octyl gallate	Aluminum oxide	W/O, *in situ* hydrophobization	Sturzenegger et al., 2012
	Methyl orange	Layered double hydroxide	O/W, enhanced emulsion stability, *in situ* confocal fluorescence microscopic images at interface	Li et al., 2013
	Fatty Acids	CaCO_3_	O/W or W/O, switchable Pickering emulsions depending on the absorbed amount of amphiphile	Cui et al., 2012
	CTAB and SDS	Silica	O/W; switchable Pickering emulsions with the sequential addition of oppositely charged surfactants	Zhu et al., 2015
	Cationic surfactants	Silica	O/W, W/O; double phase inversion achieved with two-tails cationic surfactants	Cui et al., 2010
	*N′*-dodecyl-*N,N*-dimethylacetamidine	Silica	O/W; switchable Pickering emulsion triggered by CO_2_ responsive surfactant	Jiang et al., 2013
	PDMAEMA-*b*-PMMA	PS latex nanoparticle	O/W, W/O; switchable Pickering emulsion triggered by temperature responsive PDMA blocks	Binks et al., 2005
	PEI	Silica	O/W, W/O, W/O/W; the emulsion type can be easily tuned by the adsorbed amount of PEI	Williams et al., 2014a,b
	PLLA	HAp	O/W, biocompatible microspheres, *in situ* modification at interfaces	Fujii et al., 2009
	PS	HAp	O/W, *in situ* modification at interfaces	Okada et al., 2012
	Amine end-capped PDMS	Carboxylated PS nanoparticles	O/W; *in situ* modification at interfaces, interfacial jamming	Cui et al., 2013
	Amine end-cappd PS	Cellulose nanocrystal	W/O; *in situ* modification at interfaces, liquid tubule	Liu et al., 2017
	PDMS	Carboxylated PS nanoparticles	O/W/O, W/O, bicontinuous jammed emulsions	Huang et al., 2017
Chemical anchoring	Silane coupling agents	Fe_3_O_4_ nanoparticles	O/W; stable magnetic Pickering emulsions	Zhou et al., 2012
	Carboxyl containing spiropyran	UCNP@SiO_2_	W/O, O/W; switchable Pickering emulsions triggered by NIR/visible light, interfacial catalysis	Chen et al., 2014
	organosilanes	Silica	O/W; switchable Pickering emulsions triggered by pH	Yang et al., 2013
	PNIPAM	Cellulose nanocrystals	O/W; thermoresponsiveness of Pickering emulsions	Zoppe et al., 2012
	PMETAC	Silica	O/W; ion-specific responsive Pickering emulsions	Tan et al., 2011
	*N,N*-dimethylacetamidine	Silica	O/W, W/O; switchable Pickering emulsions triggered by CO_2_	Liang et al., 2014
	thiol-terminated PEG chains and short alkane-thiol molecules	Gold	O/W; emulsions with enhanced stability stabilized by gold particles with both hydrophilic and hydrophobic chains	Larson-Smith and Pozzo, 2012
	hydrophilic & hydrophobic organosilanes	Silica	O/W; particles modified by both hydrophilic and hydrophobic groups generate emulsions with highest stability	Björkegren et al., 2017
	Sulfonated PS	Silica	Double emulsions; interfacial catalysis for biphasic reactions	Shi et al., 2015
	PS-*b*-P2VP-*b*-PEO	Silica	O/W, W/O; switchable Pickering emulsions triggered by pH	Motornov et al., 2007
	μ-PEG-*b*-PS-*b*-PIPSMA	Silica	O/W, W/O, O/W/O; tuning the wettability of the modified nanoparticles by solvent environment and host-guest complexation, initial location effect and one-step fabrication of multiple emulsions	Liu et al., 2016
Roughness	Eudragit S100 nanoparticles	Amine modified silica particles	O/W; surface roughness benefits emulsions stability in Wenzel regime and destabilize emulsion in Cassie-Baxter regime	San-Miguel and Behrens, 2012
	Negatively charged silica nanoparticles	Positively charged silica microparticles	O/W, W/O; initial location effect	Zanini et al., 2017

*CTAB, cetyltrimethylammonium bromide; SDS: sodium dodecyl sulfate; PDMAEMA-b-PMMA: poly(2-(dimethylamino)ethyl methacrylate)-b-poly(methyl methacrylate); PEI, poly(ethylene imine); PLLA, poly(L-lactic acid); Hap, hydroxyapatite; PS, polystyrene; PDMS, polydimethylsiloxane; PMETAC, poly((2-(methacryloyloxy)ethyl)- trimethylammonium chloride); Poly(N-isopropylacrylamide): PNIPAM; PS-b-P2VP-b-PEO: polystyrene-b-poly(2-vinylpyridine)-b-poly(ethylene oxide); μ-PEG-b-PS-b-PIPSMA: μ-poly(ethylene glycol)-b-polystyrene-b-poly[(3-triisopropyloxysilyl)propyl methacrylate] miktoarm star terpolymers; Eudragit S100 nanoparticles: poly(methacrylic acid-co-methyl methacrylate) nanoparticles, 50 nm*.

## Tailoring the wettability by surface chemistry

Particle surface chemistry regulates particle retention, wettability alteration, ability to stabilize emulsions and foams. There are two main approaches developed for adjusting the surface chemistry for controllable wettability of particles: physical adsorption and chemical anchoring of small molecules or polymers.

### Surface modification by physical adsorption

**Figure 1:**
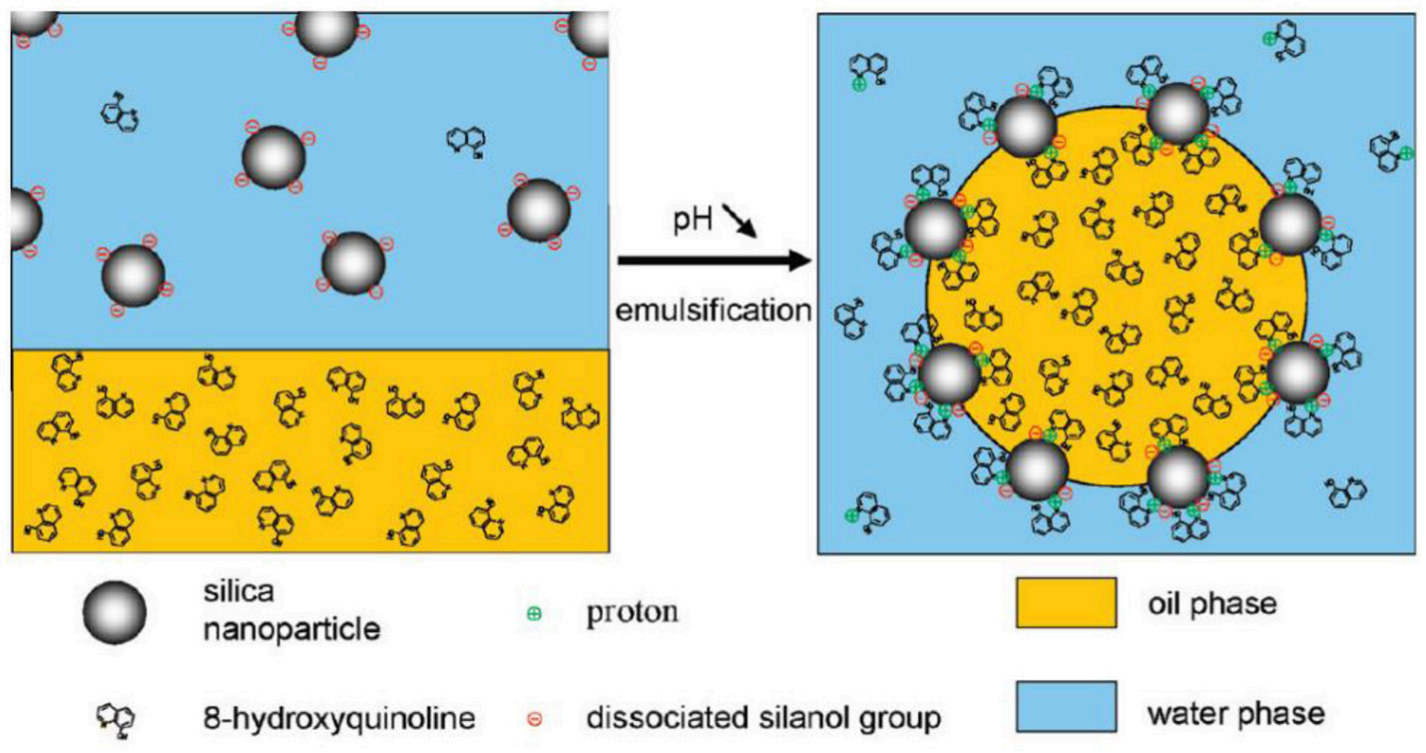
Schematic representation of pH-sensitive Pickering emulsion stabilized by silica nanoparticles and hydrophobizing agent 8-hydroxyquinoline (8-HQ) (Haase et al., 2010). Reprinted with permission from Haase et al. (2010). Copyright 2010 American Chemical Society.

Surface modification by physiosorbed small molecules has attracted significant interest due to its simple preparation and efficacy. The pristine nanoparticles are typically too hydrophilic to form stable emulsion. The adsorption of surface inactive small molecules can efficiently enhance the hydrophobicity of nanoparticles, so that the modified particles preferentially stay at the oil/water interfaces and correspondingly bring out stable emulsion. Frith and coworkers explored the trivalent La^3+^ cations to tune the hydrophilicity of negatively charged silica nanoparticles (Frith et al., 2008). The adsorption of La^3+^ onto the silica particles reduce their effective charge and synergistically promote to form stable O/W Pickering emulsions. Also, Hasse and coworkers revealed that the adsorption of 8-hydroquinoline (8-HQ) onto the surface of hydrophilic silica particles rendering them hydrophobicity and therefore more interfacially active, as shown in Figure 1 (Haase et al., 2010). O/W Pickering emulsions with dispersed diethyl phthalate droplets containing 8-HQ stabilized by 20 nm Ludox TMA silica nanoparticles were stable in a narrow pH range from 4.4 to 5.5 (Haase et al., 2010). Above pH 5.5, only small fractions of 8-HQ was protonated and dissolved in aqueous phase. Because of this, insufficient adsorption of protonated 8-HQ onto silica particles leads to unstable emulsions. In contrast, excess adsorption of protonated 8-HQ below pH 4.4 forms bilayer structure on silica particles, which also causes emulsion destabilization. Binks and coworkers demonstrated that the inherently hydrophilic particles can be hydrophobilized *in situ* by the adsorption of dissolved oil molecules (dialkyl adipate) in the aqueous phase and enable them to stabilize O/W emulsions (Binks and Yin, 2016). This kind of surface modification method is mainly dependent on the solubility of dialkyl adipate in water, which can be systematically adjusted by altering the chain length of alkyl group. Based on adsorption of palmitic acid (PA) onto silica nanoparticles, Santini and coworkers pointed out that emulsions stability is associated with the hydrophobicity of nanoparticles-surfactant complexes, in which the surfactant concentration and the volume ratio between the dispersed liquids are extremely important. The most stable emulsions can be produced only when surfactant molecules form single layers at the particle surface (Santini et al., 2014). By optimizing the parameters, submicrometer-sized Pickering emulsions were produced with silica nanoparticles and adsorbed oleic acid for a wide range of oils (Sadeghpour et al., 2013). Similar results have also been reported for the surface modification of other inorganic nanoparticles such as Laponite, layered double hydroxide (LDH), aluminum oxide and clay through electrostatic attraction or hydrogen bonding interaction (Li et al., 2012, 2013; Reger et al., 2012; Sturzenegger et al., 2012).

**Figure 2:**
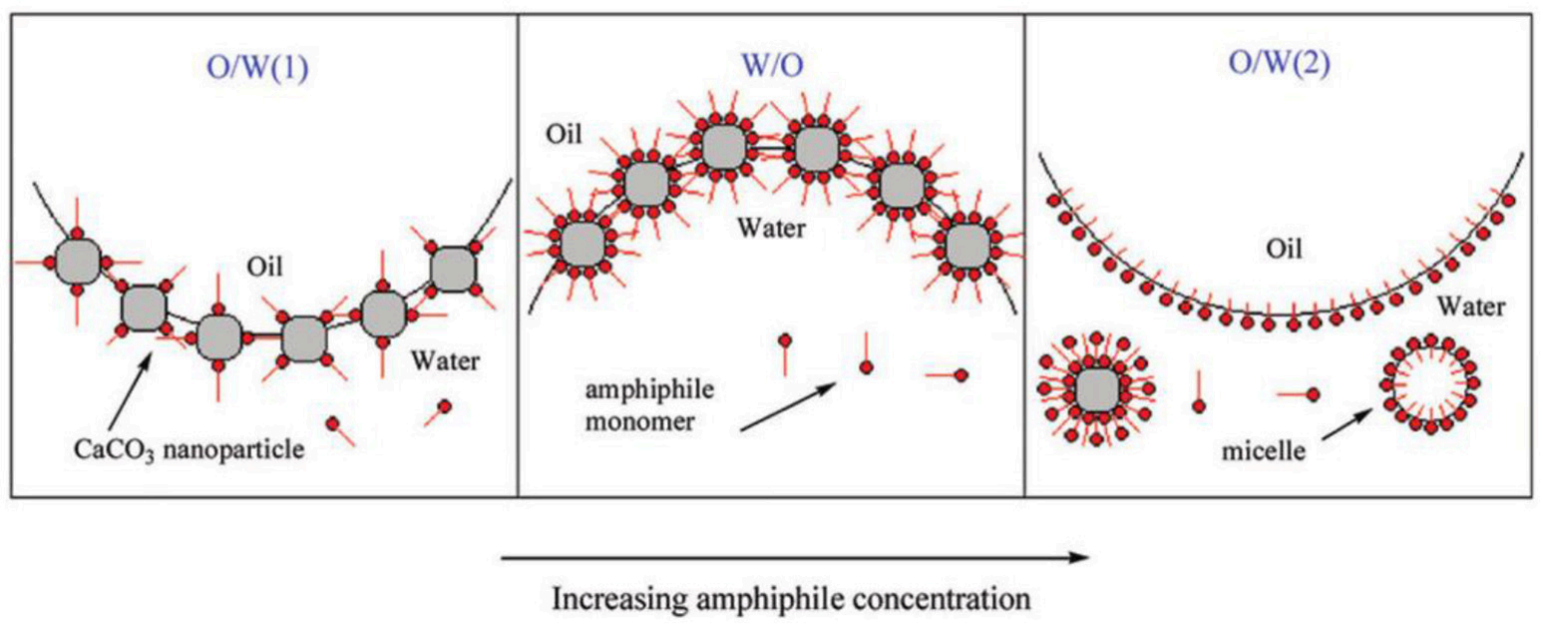
Schematic representation of phase inversion induced by the adsorption of sodium carboxylates onto CaCO_3_ nanoparticles (Cui et al., 2012). Reprinted with permission from Cui et al. (2012). Copyright 2012 American Chemical Society.

Besides interfacially inactive small molecules, the adsorption of surfactants onto colloidal particles can also synergistically enhance the formation of Pickering emulsions. Yuan and coworkers proposed two distinct co-stabilization mechanisms that arise from interactions between the nanoparticles and surfactant molecules (Yuan and Williams, 2016). Generally, significant interaction generate faster wetting process for nanoparticles at the oil/water interface and yields enhanced stabilization. On the contrary, competitive adsorption of nanoparticles and surfactant molecules occurs to stabilize the droplets when significant interaction is not existed. The adsorption/desorption equilibrium between surfactant molecules regulates the droplet stability. Cui and coworkers demonstrated that the *in situ* surface modification of pristine CaCO_3_ nanoparticles by the adsorption of a series of sodium carboxylates could stabilize Pickering emulsions (Cui et al., 2012). The negatively charged headgroups of sodium carboxylates can absorb onto the positively charged CaCO_3_ nanoparticles and form a monolayer, resulting in enhanced hydrophobicity of the particle surface. When the particle surface is modified to a particular hydrophobicity, emulsion type inversed from O/W(1) to W/O as shown in Figure 2. As for sodium dodecanoate, a second phase inversion from W/O to O/W(2) emulsions occurred at high amphiphile concentration. This phase inversion was attributed to the bilayer or hemimicelle formation of sodium dodecanoate at high concentration, which endowed the particle surface hydrophilicity again and induced desorption of the particles from the interface. Consequently, the emulsion droplets were stabilized solely by the surfactants (Cui et al., 2012).

The switchable Pickering emulsions were reported with negatively charged silica nanoparticles and the consequent addition of cationic and anionic surfactants (Zhu et al., 2015). The *in situ* hydrophobization of silica nanoparticles with cetyltrimethylammonium bromide (CTAB) enable them to stabilize stable O/W emulsions. The destabilization of emulsions can be triggered by adding an equimolar amount of sodium dodecyl sulfate (SDS) because of the stronger electrostatic interaction between cationic and anionic surfactants. The same authors further investigated the effect of the architecture of the ionic surfactants on the *in situ* hydrophobization of the oppositely charged particles (Cui et al., 2010). They found that using single-chain trimethylammonium bromide surfactants or a double-head gemini cationic surfactant, only O/W emulsions can be prepared. In contrast, using didodecyldimethylammonium bromide with double alkyl tail-group, the hydrophobicity of silica particles is sufficient to stabilize W/O emulsions, and phase inversion from O/W to W/O occurred. Jiang and coworkers have also prepared a switchable Pickering emulsion by using a combination of silica nanoparticles and a trace amount of switchable surfactant *N'*-dodecyl-*N,N*-dimethylacetamidine (Jiang et al., 2013). Upon the addition of CO_2_, the neutral amidine group converts to cationic amidinium group, which adsorbed onto the silica particles and then stable *n*-octane-in-water emulsion were prepared with the *in situ* formed nanoparticle surfactant. Demulsification of the as-prepared Pickering emulsion could be achieved by bubbling N_2_.

Compared to small molecules, anchoring polymer chains onto particles by physical adsorption or chemical bonding makes the particles extremely efficient Pickering emulsion stabilizers (Binks et al., 2005; Saleh et al., 2005a; Saigal et al., 2010; Isa et al., 2011). Generally, pristine particles do not reduce the interfacial tension, whereas grafted polymer chains reduce the interfacial tension by penetrating the oil/water interface and (Saigal et al., 2010). Furthermore, the desorption of a particle grafted with polymer corresponds to the simultaneous detachment of many polymer chains from oil/water interfaces. For this kind of emulsifiers, the solubility of the grafted polymer chains in oil and water may significantly influence the emulsion characteristics.

**Figure 3:**
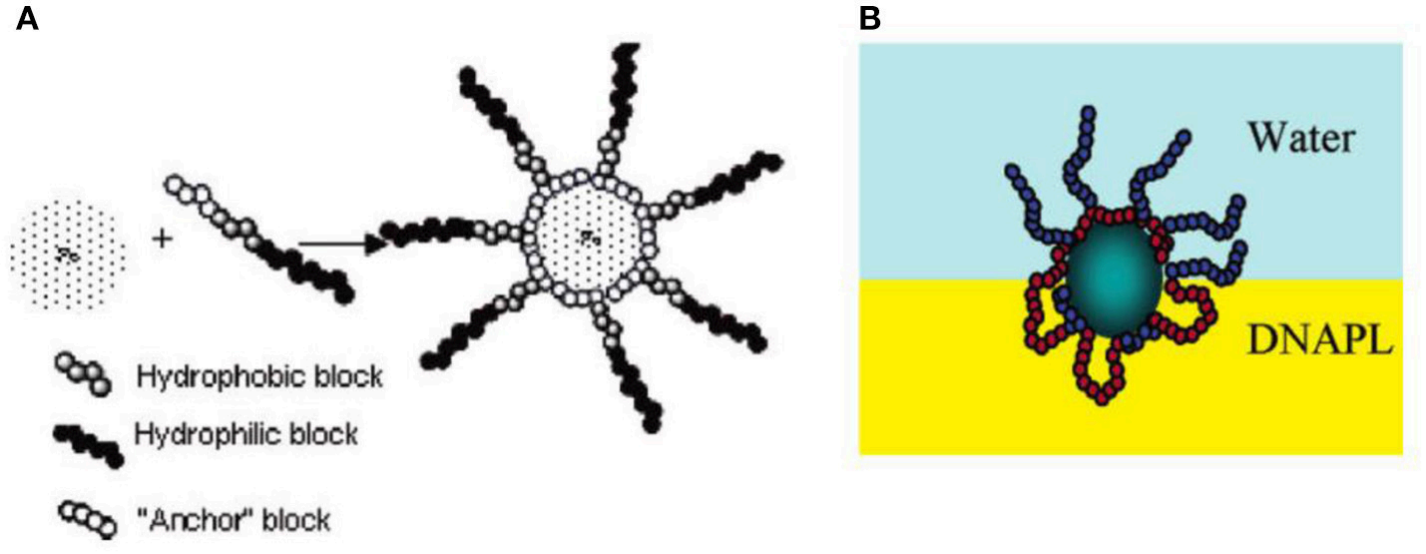
Schematics representation of triblock copolymer-modified iron nanoparticles **(A)** and proposed chain rearrangement at oil/water interface **(B)**, the oil phase is toxic non-aqueous phase liquids (NAPL) (Saleh et al., 2005a). Reprinted with permission from Saleh et al. (2005a). Copyright 2005 American Chemical Society.

Lucio and coworkers have demonstrated that surface modification with nitro catechol-endcapped polyethylene glycol (PEG) endows Fe_3_O_4_ nanoparticles with superior stability. They showed that the thermodynamics adsorption of the functionalized particles at water/n-decane interface is dominated by the solubility of PEG in each phase (Isa et al., 2011). Binks and coworkers have employed poly(2-(dimethylamino)ethyl methacrylate-*b*-poly(methyl methacrylate) (PDMAEMA-*b*-PMMA) stabilized polystyrene latex nanoparticles to prepare thermos-responsive Pickering emulsions (Binks et al., 2005). Hydrophobic PMMA block acts as the anchoring block and hydrophilic PDMAEMA acts as steric stabilizer. The wettability of the particles can be tailored by temperature because of thermos-responsive properties of PDMAEMA block, which resulted in the phase inversion from O/W below the lower critical solution temperature (LCST) and to W/O above the LCST. Saleh and coworkers have reported that physisorbed layers of amphiphilic poly(methacrylic acid)-*b*-poly(methyl methacrylate)-*b*-poly(styrene- sulfonate) (PMAA-*b*-PMMA-*b*-PSS) triblock copolymers can improve the stability of Fe^0^/Fe_3_O_4_ nanoparticles in water and render them amphiphilicity at the oil/water interfaces via chain rearrangement, as shown in Figure 3 (Saleh et al., 2005a). The adsorption of copolymer-modified iron nanoparticles at oil/water interfaces and the formation of O/W Pickering emulsions have potential application in the remediation of chlorinated organic-contaminated groundwater.

**Figure 4:**
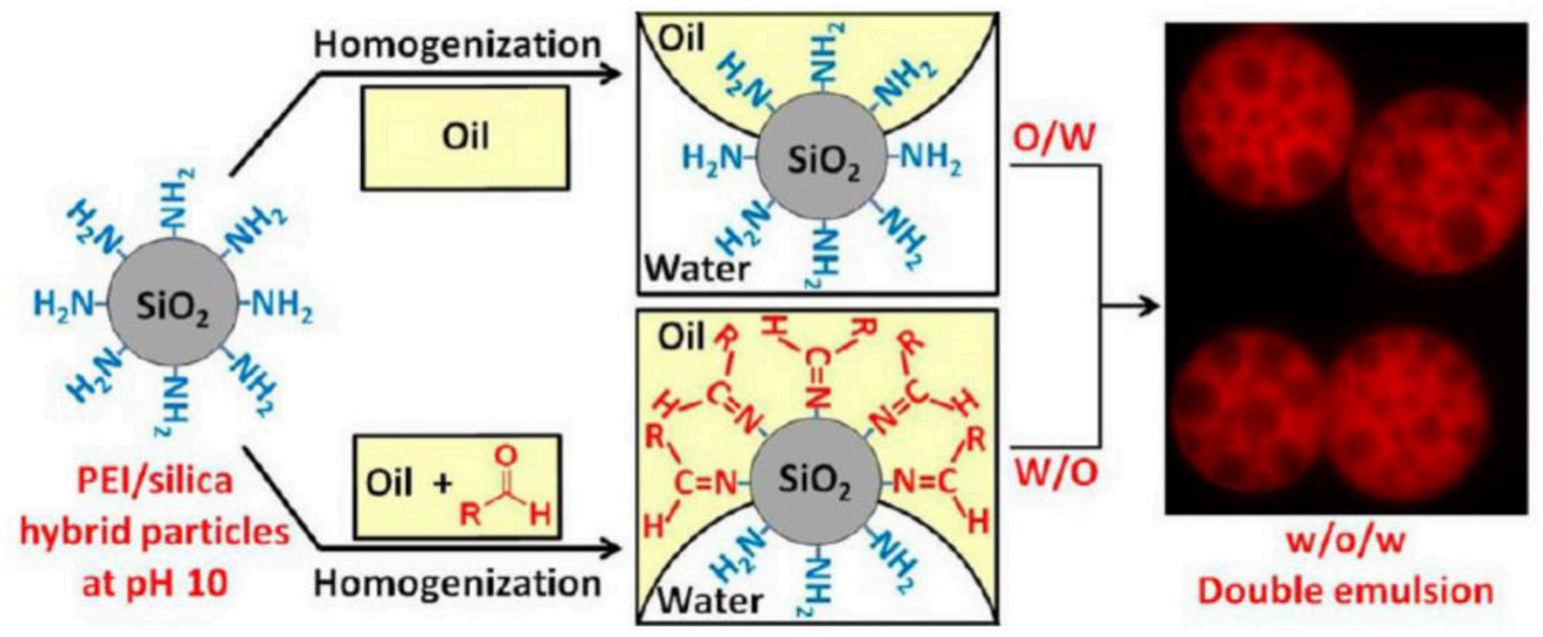
Schematic illustration of the preparation of W/O/W multiple emulsions using hybrid PEI/silica particles with adjustable wettability via Schiff base chemistry (Williams et al., 2014b). Reprinted with permission Williams et al. (2014b). Copyright 2014 American Chemical Society.

It has been demonstrated that poly(ethylene imine) (PEI) can be physisorbed onto fumed silica particles to tune their surface wettability (Williams et al., 2014a,b). The wettability of such hybrid PEI/silica particles can be easily tailored by changing the PEI/silica weight ratio (Williams et al., 2014a). Systematic variation of the PEI/silica weight ratio induces the phase inversion from O/W to W/O emulsions. Stable water-in-oil-in-water (W/O/W) multiple emulsions can be prepared with the combination of two kind of PEI/silica hybrid particles with mass ratios of 0.075 and 0.50 at pH 10, respectively. Therefore, the hydrophilic hybrid particles with mass ratio of 0.075 stabilized the O/W interface and the hydrophobic hybrid particles with mass ratio of 0.50 stabilized the W/O interface. The authors further demonstrated the wettability of PEI/silica hybrid particles could be tailored by *in situ* Schiff base chemistry between primary or secondary amine groups of PEI and long chain aldehyde (1-undecanal) in oil phase, as shown in Figure 4 (Williams et al., 2014b). This interfacial reaction increases the hydrophobicity of PEI/silica hybrid particles. In accordance to the surface wettability of hybrid particles, various type of Pickering emulsions can be obtained including O/W, W/O, and W/O/W emulsions.

**Figure 5:**
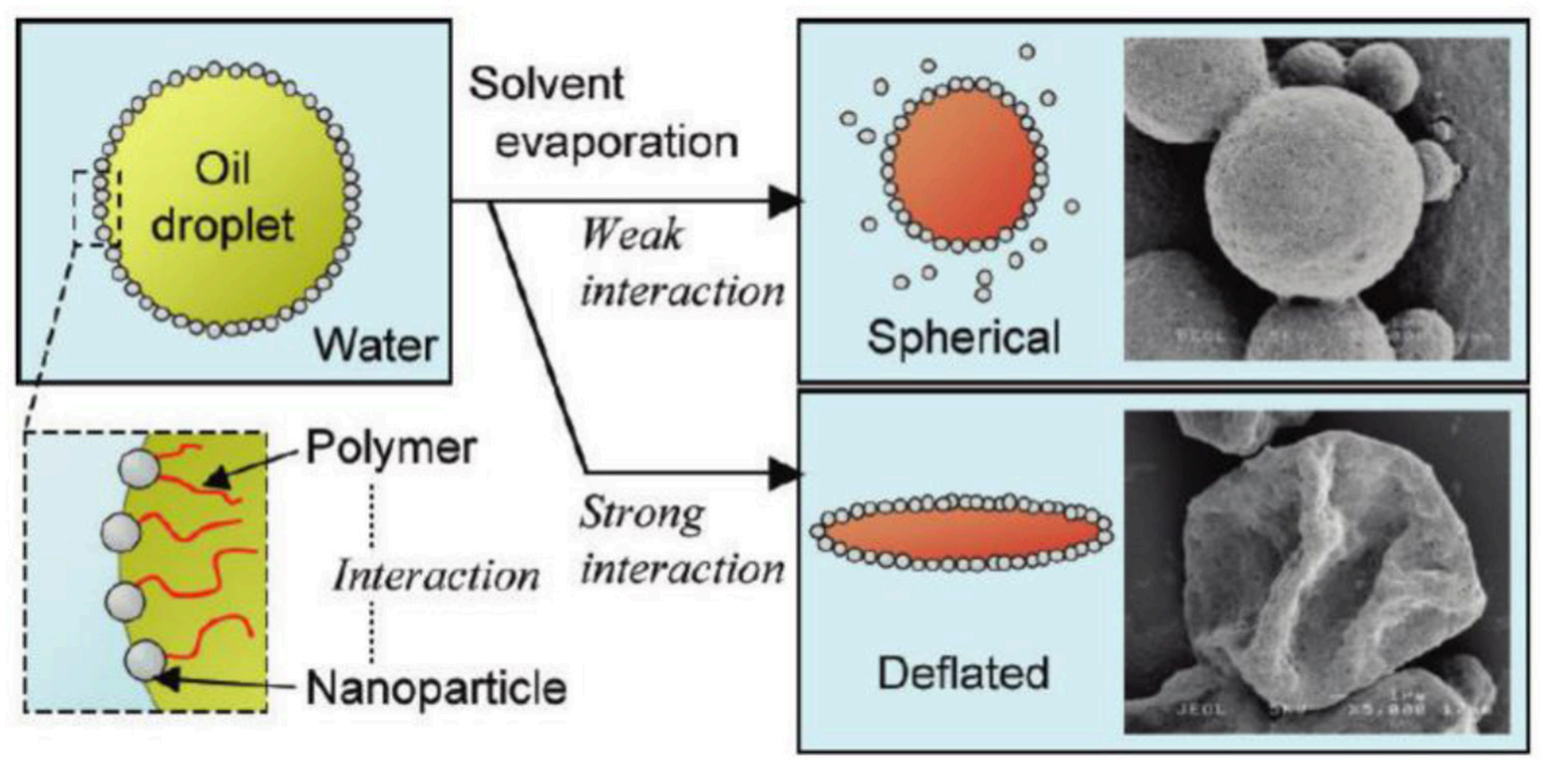
Schematic representation of O/W emulsion stabilized by the combination of hydroxyapatite (HAp) particles in aqueous solution and polystyrene containing end groups in oil phase; and the effect of interaction between HAp particles and polymer on the morphology of microparticles after evaporation of oil phase (Okada et al., 2012). Reprinted with permission from Okada et al. (2012). Copyright 2012 American Chemical Society.

Fujii and coworkers found that hydroxyapatite (HAp) stabilized O/W emulsions were prepared only for the oils containing ester group such as methyl myristate (Fujii et al., 2007). They further demonstrated that dichloromethane-in-water emulsions could be stabilized by HAp particles when poly(L-lactic acid) (PLLA) was dissolved in oil phase (Fujii et al., 2009). Instead of the interactions between HAp and ester groups of polymer chain at the interface, Okada and coworkers prepared O/W Pickering emulsions stabilized by the combination of HAp particles and polystyrene containing end groups in oil phase (Okada et al., 2012). The driving force for the formation of the stable O/W emulsions is the interaction between HAp particles and carboxyl group of polystyrene at oil/water interfaces. After evaporation of dichloromethane for the emulsions, HAp particles coated microspheres were fabricated as shown in Figure 5 (Okada et al., 2012). When weak interactions are involved, HAp particles may desorb from the interface during the evaporation of dichloromethane and shrinkage of the interface. However, strong interactions between HAp particles and polymer in oil phase prevent desorption of HAp particles from the interface, resulting in deflated microspheres.

Cui and coworkers detailly investigated the interfacial jamming of nanoparticles at the oil/water interfaces with the aid of end-functionalized polymer chains in oil phase (Cui et al., 2013). Aqueous dispersion of 15 nm carboxylated polystyrene (PS-COOH) nanoparticles was dispersed in silicone oil containing amine end-capped polydimethylsiloxane. The water droplets were stabilized by the nanoparticle surfactants formed *in situ* at the interface via the carboxylate-amine interactions. It is worth noting that neither PS-COOH particles nor functionalized polydimethylsiloxane (PDMS) alone is interfacially active to arrest the coalescence of water droplets. Under the external electric field, the water droplets could deform from spherical to ellipsoidal shapes. Consequently, more nanoparticles surfactants were formed at the interface because of the increase of the oil/water interface from the deformation. More interestingly, the water droplets maintained the deformed shape stabilizing by the interfacially jammed nanoparticle surfactants after the removal of the electric field. Based on the same principle, Liu and coworkers described the interfacial activity of nanoparticle surfactants formed at the toluene/water interface with rod-like cellulose nanocrystal (CNC) dispersed in water and amine end-capped polystyrene in toluene (Liu et al., 2017). Under the optimized experimental conditions, the formation of CNC-surfactants at the interface can arrest the Rayleigh instability and generate aqueous tubules in toluene.

Previously, bicontinuous jammed emulsions (bijels) were mainly produced through spinodal decomposition process and the formed two continuous, interconnected domains were stabilized by particles with equal affinity with the two liquids (Stratford et al., 2005; Herzig et al., 2007). Recently, Huang and coworkers fabricated bijels with sub-micrometer domains using nanoparticle surfactants (Huang et al., 2017). It was found the combination of 15 nm PS-COOH particles with low molecular weight PDMS (1,000 g/mol) tends to produce either oil-in-water-in-oil (O/W/O) multiple emulsions or W/O emulsions, the combination of PS-COOH particles with high molecular weight PDMS tends to produce W/O emulsions. By contrast, bijels were produced by the combination of PS-COOH particles with a mixture of low and high molecular weight PDMS.

**Figure 6:**
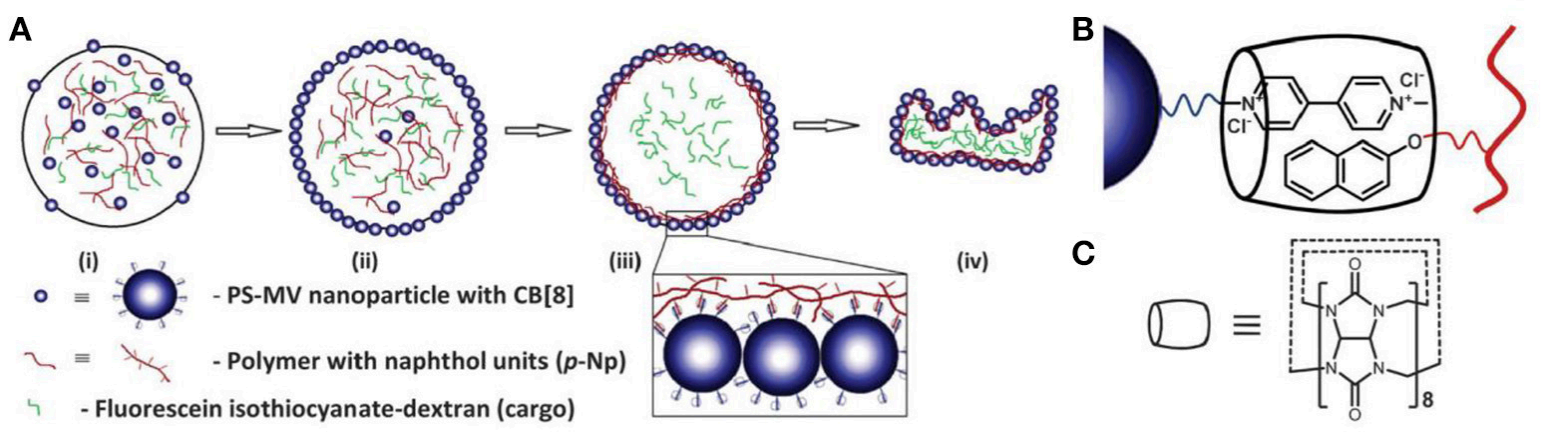
Schematic representation of supramolecular colloidosomes through the interfacially host-guest crosslinking between cucurbi[8]uril (CB[8]), methyl viologen-functionalized polystyrene nanoparticles and naphthol-functionalized polyacrylamide (Stephenson et al., 2014). **(A)** Schematic of colloidosome formation. **(B)** Schematic of the ternary supramolecular complex formed between PS-MV, p-Np and CB[8]. **(C)** The molecular structure of CB[8]. Reprinted with permission from Stephenson et al. (2014). Copyright 2014 The Royal Society of Chemistry.

In addition to the electrostatic interaction, supramolecular complexation between particles and modifiers has also been employed in assembling supramolecular colloidosomes at the oil/water interface (Mathapa and Paunov, 2013; Stephenson et al., 2014). Mathapa and coworkers have reported that O/W Pickering emulsion can be stabilized by the *in situ* formed inclusion complex of cyclodextrin (CD) and tetradecane. Similarly, W/O emulsions can be formed when highly viscous silicone oil was used as continuous phase (Mathapa and Paunov, 2013). Stephenson and coworkers have explored the cucurbi[8]uril (CB[8]) host-guest system to interfacially crosslink methyl viologen-functionalized polystyrene nanoparticles by naphthol-functionalized polyacrylamide as shown in Figure 6 (Stephenson et al., 2014). Triggered release of cargoes from the supramolecular colloidosomes has been demonstrated by disassembly of the supramolecular complex with the addition of competitive guest such as 1-adamanthlamine (ADA) for CB[8] under mild condition.

Besides, temperature induced phase separation was applied to tune the wettability of hybrid particles of polymer@silica (Fuchs and Avnir, 2013). Under thermal treatment, most of the entrapped hydrophobic polymers emerge at particles surface and endow the hybrid particles with amphiphilic properties for stabilizing W/O or O/W emulsions (Fuchs and Avnir, 2013). Zhou and coworkers designed a thermally switched release system consisted of aqueous colloidosomes stabilized by β-cyclodextrin (β-CD) nanoparticles (Zhou et al., 2013). Pluronic L31, the poly(ethylene oxide)-*b*-poly(propylene oxide)-*b*-poly(ethylene oxide) (PEO-*b*-PPO-*b*-PEO) triblock copolymer, was dissolved in the inner aqueous phase with 6.0 wt%. The release of active materials was triggered by the temperature change. Under ambient temperature (21°C), the interstitial pores were blocked by the adsorption of PEO-*b*-PPO-*b*-PEO triblock copolymers and then inhibited the release of active materials from inner to outer aqueous phase. By contrast, at 37°C, PEO-*b*-PPO-*b*-PEO triblock copolymers self-assembled into micelles and the pores were opened by the desorption of the triblock copolymers.

### Surface modification by chemical anchoring

It was demonstrated that silane coupling agents-modified Fe_3_O_4_ nanoparticles can stabilize emulsions with both non-polar oil (dodecane) and polar oil (butyl butyrate) (Zhou et al., 2012). The hydrophobicity of Fe_3_O_4_ nanoparticles increased with the modification by either fatty acids or silane coupling agents with different chain lengths. However, fatty acids-coated Fe_3_O_4_ nanoparticles can only stabilize emulsions containing non-polar oil and are incapable to stabilize emulsions composed of butyl butyrate. Thermal gravimetric analysis indicates that the adsorbed amount of carboxylic acid via physical adsorption is less than silane coupling agents via chemical anchoring (Zhou et al., 2012).

**Figure 7:**
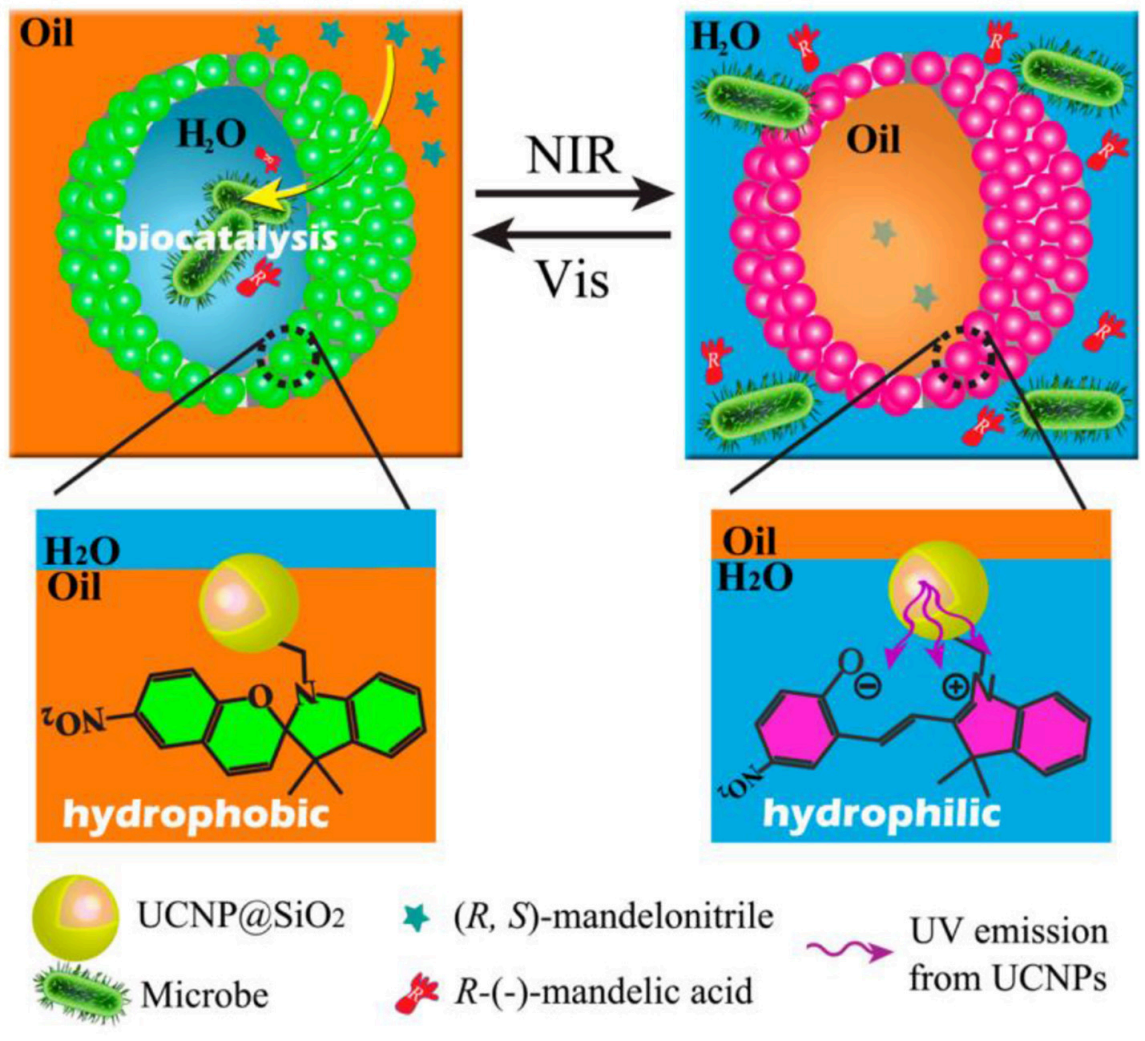
Schematic representation of NIR/visible light controlled Pickering emulsions for biphasec enantioselective biocatalysis (Chen et al., 2014). Reprinted with permission from Chen et al. (2014). Copyright 2014 American Chemical Society.

A novel NIR/visible light controlled Pickering emulsion stabilized by photochromic spiropyran conjugated upconversion nanophosphors (UCNPs) was developed and applied for biocatalytic applications as shown in Figure 7 (Chen et al., 2014). Under NIR excitation, the UCNPs emit UV light and induce the formation of hydrophilic open-ring form of spiropyran. This isomerization process can be reversed by exposure to visible light. Light-triggered emulsion inversion was attributed to the surface switchable of UCNPs. Based on this intelligent Pickering emulsion, catalytic performance was enhanced, and substrate inhibition effect was relieved (Chen et al., 2014).

Yang and coworkers fabricated pH-responsive and interfacially active modified silica particles with a mixture of hydrophobic (CH_3_O)_3_Si(CH_2_)_7_CH_3_ and hydrophilic, pH sensitive (CH_3_O)_3_SiCH_2_CH_2_CH_2_(NHCH_2_CH_2_)_2_NH_2_ organosilanes by covalent linkage via sol-gel chemistry (Yang et al., 2013). After loading catalysts, the functionalized silica particle-stabilized emulsions show higher catalysis efficiency than its analogous biphasic systems. More interestingly, the separation and recycling of sub-micrometer solid catalyst can be achieved by changing the pH value based on pH-triggered emulsion phase inversion (Yang et al., 2013).

**Figure 8:**
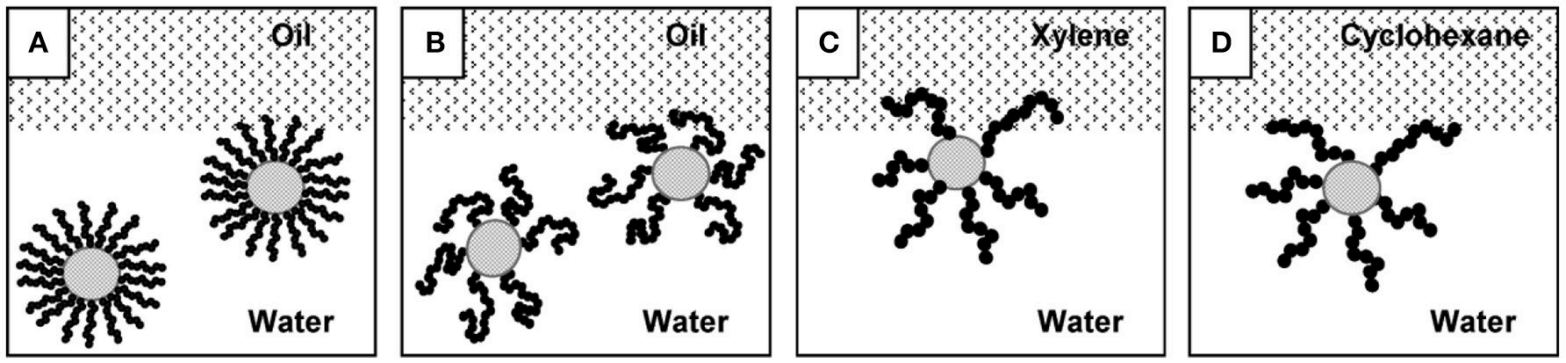
Effect of grafting density and the solubility of the grafted polymer chain in oil and water phases on the location of the hybrid silica nanoparticles (Saigal et al., 2010). Particles with high grafting density **(A)** are restricted in their ability to reorganize and penetrate the interface compared to particles with a low grafting density **(B)**. Proposed configurations of a SiO_2_-PDMAEMA particle at an oil/water interface, with chains able to penetrate xylene **(C)**, but not cyclohexane **(D)**. Reprinted with permission from Saigal et al. (2010). Copyright 2010 American Chemical Society.

Tilton and coworkers investigated the Pickering emulsions stabilized by thermally responsive polymer grafted silica nanoparticles (Saigal et al., 2010). Poly(2-(dimethylamino)ethyl methacrylate) (PDMAEMA) chains displaying pH- and thermo-responsive behaviors, were grafted from 20 nm silica particles with controlled chain length and grafting density by atom-transfer radical polymerization (ATRP). These functionalized silica particles can be used to form highly stable O/W emulsions at extremely low concentrations. Emulsions prepared according to this method were thermos-responsive, and rapidly demulsified upon increasing the temperature above the critical flocculation temperature (CFT) of the SiO_2_-PDMAEMA particles. The lowest grafting density particles were observed to be more efficient and robust emulsifiers than high grafting density particles, which stemmed from the chain configurational freedom of the grafted polymer chain as shown in Figure 8 (Saigal et al., 2010). For the lowest grafting density particles, these particles can emulsify both the xylene (a good solvent for PDMAEMA) and cyclohexane (a poor solvent for PDMAEMA) below and above the CFT. However, for high grafting density particles, although the solvent quality of oil phase had no influence below CFT, it was an important factor above CFT. At 70°C, emulsions were formed with cyclohexane but not with xylene. Thermo-responsive Pickering emulsions were also prepared with cellulose nanocrystals (CNCs) grafted with poly(*N*-isopropylacrylamide) (PNIPAM) brushes (Zoppe et al., 2012). The emulsions were observed to be stable at a temperature below the LCST of PNIPAM. It was proposed that the partial collapse of PNIPAM brushes above LCST rendered the nanoparticle surfaces less hydrophilic and produced less stable emulsion.

Saigal and coworkers investigated the interactions between poly(2-(dimethylamino)ethyl methacrylate) (PDMAEMA)-grafted silica nanoparticles and three kinds of molecular surfactants, the anionic surfactant SDS, the non-ionic water-soluble surfactant Triton X-100 and the non-ionic oil-soluble surfactant Span 85, respectively (Saigal et al., 2015). Emulsification efficacy was improved with SDS at low concentrations range, where particle absorption is enhanced due to the complexation between PDMAEMA and SDS. At high SDS concentration, the SDS at interface repel the SiO_2_-PDMAEMA-SDS complexes due to charge reversal. Synergism was also observed for Triton-100, while Span 85 had no observable effect on the emulsification efficacy.

Stable O/W emulsions can also be stabilized by silica nanoparticles grafted with highly charged polyelectrolyte (Saleh et al., 2005b). Fully sulfonated poly(styrenesulfonate) (PSS) were grafted from silica by ATRP. The emulsifying effectiveness of such modified particles was ascribed to the hydrophobic vinylic polymer backbone, which rendered this highly charged polyelectrolyte surface interfacially active. Similarly, cationic poly (2-(methacryloyloxy)ethyltrimethyl- ammonium chloride) (PMETAC) brushes were grafted from 320 nm silica nanoparticles by ATRP, which can be used to produce stable O/W Pickering emulsions (Tan et al., 2011). PMETAC brushes show ion-specific collapse behavior, which induce the switching of surface hydrophilicity. The resulting colloidal dispersion was responsive to perchlorate ions (ClO^4−^), which triggered particle aggregation and then enabled the formation of emulsions. The formation of stable emulsions was not simply due to brush collapse but also due to shielding of electrostatic repulsion.

When two kinds of agents were grafted onto particle surface, the wettability can be easily controlled by the balance between hydrophilic and hydrophobic components on the surface. Liang and coworkers showed that silica particles with only CO_2_-responsive groups are capable of stabilizing O/W emulsions, while particles grafted with both CO_2_-responsive and hydrophobic chemical functional groups are capable of stabilizing W/O emulsions (Liang et al., 2014). Larson-Smith and coworkers modified gold nanoparticles with thiol-terminated polyethylene glycol (PEG) and alkane-thiol molecules (Larson-Smith and Pozzo, 2012). The resulting nanoparticles showed effective stabilization for emulsions because of strong adsorption at oil/water interfaces. Similarly, Sanna Björkegren has prepared colloidal silica particles modified with hydrophilic PEG silane and hydrophobic organosilanes containing propyl and methyl groups for Pickering emulsions (Björkegren et al., 2017). It was determined that particles containing hydrophobic groups produced emulsions with smaller droplets and higher stability. The emulsification performance was further improved by amphiphilic groups.

Besides balance between two kinds components, the wettability of colloidal particles can also be adjusted by functionalization of the grafted surface modifiers (Shi et al., 2015). Shi and coworkers have prepared amphiphilic nanoparticles with tunable properties by sulfonation of polystyrene-grafted silica nanoparticles. The sulfonic acid centers were designed to catalyze the biphasic etherification reaction of glycerol with dodecanol at the interfaces (Shi et al., 2015). By optimizing the wettability of the particles, multiple Pickering emulsions could be produced with enhanced diffusion of glycerol and dodecanol to the acid centers.

**Figure 9:**
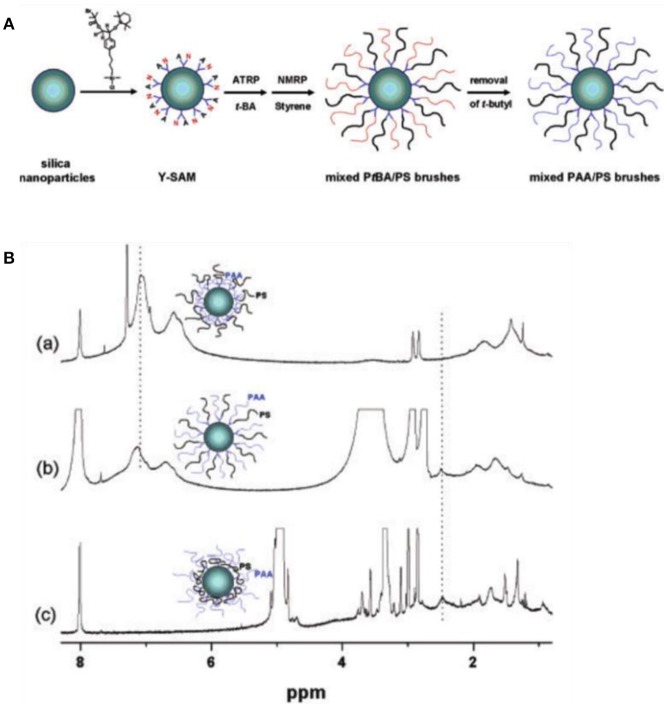
Schematic illustration of mixed polymer brushes on silica nanoparticles **(A)** and environmentally responsive properties of hairy particles in different deuterated solvent demonstrated by ^1^H NMR (Li et al., 2005). **(B)** ^1^H NMR spectra of PAA/PA particles dispersed in (a) CDCl3, (b) DMF-d7, and (c) CD3OD. Reprinted with permission from Li et al. (2005). Copyright 2005 American Chemical Society.

Recently, remarkable advances have been made in fabricating functionalized particles grafted with environmentally responsive polymer for Pickering emulsions (Zhao and Zhu, 2009). Those functionalized particles can form stable dispersion in both oil and aqueous phase with the reorganization of polymer chains based on surrounding solvent condition. Zhao and coworkers first reported the switching behavior of silica nanoparticles coated by mixed polymer brushes (Li et al., 2005). As shown in Figure 9, poly(*tert*-butyl acrylate) (P*t*BA) and polystyrene (PS) brushes were grafted from silica nanoparticles by ATRP and nitroxide-mediated radical polymerization (NMP), respectively. Subsequent hydrolysis of P*t*BA produced silica nanoparticles coated with amphiphilic poly(acrylic acid) (PAA) and PS mixed brushes. Environmentally responsive properties of the as-prepared hairy particles were demonstrated by ^1^H NMR in different deuterated solvent (Li et al., 2005). Later on, Minko and coworkers reported the application of mixed polymer brushes to tune the wettability of nanoparticles for Pickering emulsions (Motornov et al., 2007). Particles grafted with amphiphilic mixed polymer brush were prepared by quaternization reaction between bromo groups on the surface of silica particles and pyridine groups in the middle block of polystyrene-*b*-poly(2-vinylpyridine)-*b*-poly(ethylene oxide) (PS-*b*-P2VP-*b*-PEO). The wettability of functionalized particles can be precisely tuned by solvent and pH. The emulsion type is closely related to the balance between hydrophobic PS and hydrophilic P2VP and PEO in the particle surface. At pH <4, the fraction of hydrophilic components is fairly high and O/W emulsions were formed. While at pH >4, the deprotonated P2VP collapsed and contributed to the hydrophobic behavior, consequently W/O emulsions were formed. Following this central linking approach, nanoparticles grafted with poly(methyl methacrylate)-*b*-poly(glycidyl methacrylate)-*b*-polystyrene or poly(methyl methacrylate)-*b*-poly(glycidyl methacrylate)-*b*-poly(*tert*-butyl methacrylate) showed similar swithcing properties for Pickering emulsions (Cheng et al., 2008; Wang et al., 2011). The emulsion types can be tuned between O/W and W/O by altering the relative chain length of hydrophobic and hydrophilic blocks (Wang et al., 2011).

**Figure 10:**
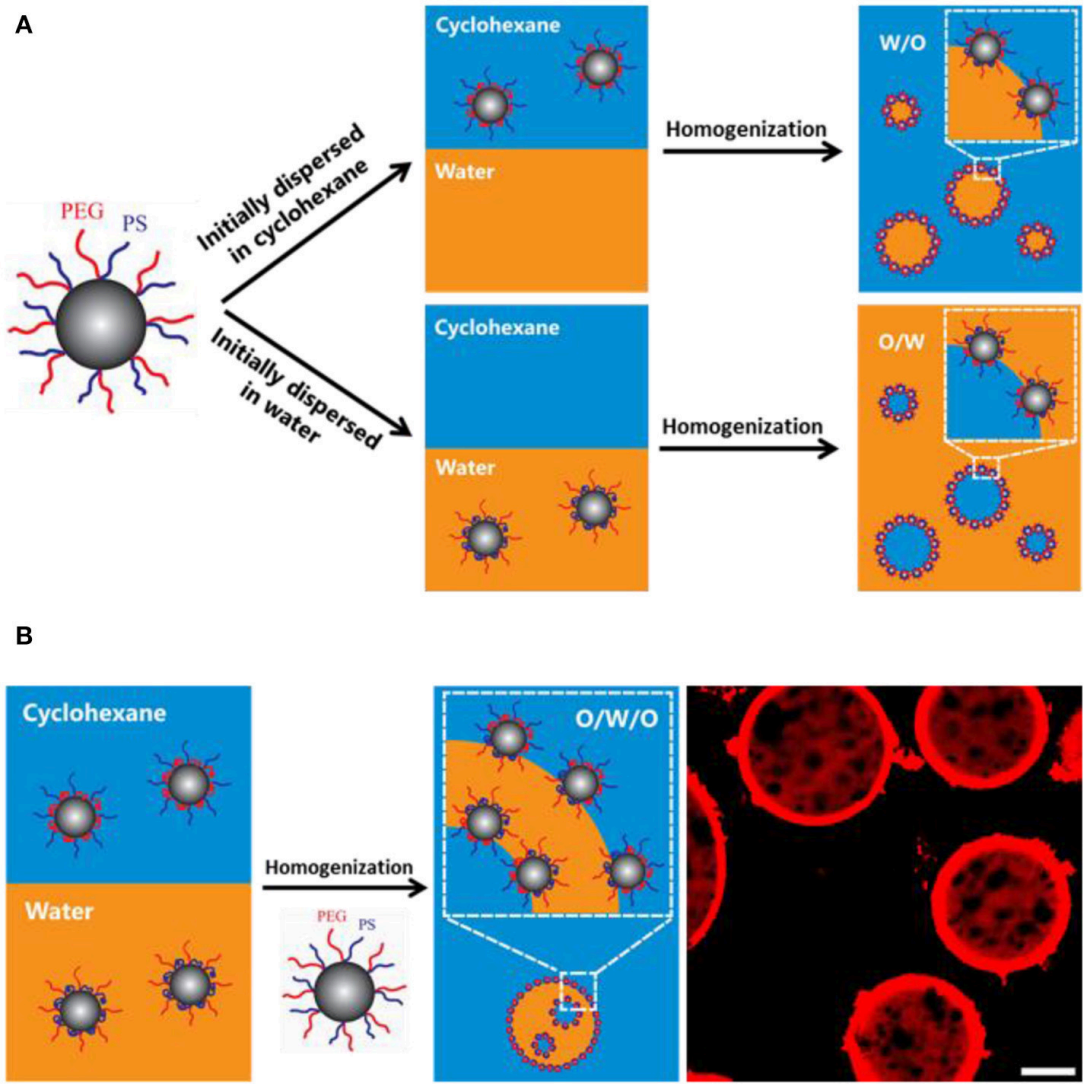
Schematic representation of the initial location of silica nanoparticles on the emulsion type **(A)** and emulsification of multiple emulsions **(B)** (Liu et al., 2016). Reprinted with permission from Liu et al. (2016). Copyright 2016 American Chemical Society.

It has been reported that amphiphilic poly(ethylene glycol)-*b*-polystyrene (PEG-*b*-PS) block copolymer can stabilize multiple emulsions via one-step phase inversion process (Hong et al., 2012). The multiple emulsions stabilized by block copolymer are highly stable and impart the ability to encapsulate both polar and non-polar cargos. The ratio of the block length, namely asymmetric ratio, greatly affects the catastrophic phase inversion as well as the emulsion type and stability (Sun et al., 2014). Recently, PEG and PS blocks have been alternately grafted onto silica nanoparticles via the sol-gel reaction between silane coupling groups of ABC miktoarm star terpolymers consisting of PEG, PS and poly[(3-triisopropyloxysilyl)propyl methacrylate] (μ-PEG-*b*-PS-*b*-PIPSMA) and silanol groups on silica surface (Liu et al., 2016). It was found that the wettability of the resulting nanoparticles can be finely tuned by the solvent environment and host-guest chemistry. The ^1^H NMR results confirmed that the wettability change is attributed to the reorganization polymer chains in different deuterated solvents. The initial location effect was shown in Figure 10. When the functionalized particles were first dispersed in oil, W/O emulsion was prepared. Also O/W emulsion was prepared when the functionalized particles were first dispersed in water. These results imply the initial location of the particles is the determining factor for the emulsion type. Inspired by the effect of initial location, multiple emulsions were fabricated with one half of the hairy particles in water, another half in oil. The hairy particle's wettability can also be tuned by supramolecular chemistry. The hydroxyl group of α-CD will increase the hydrophilicity of the hairy particle.

## Tailoring the wettability by surface roughness

In addition to the surface chemistry, the surface topology such as roughness can also affect the wetting behavior of colloidal particles and consequently affect the adsorption of particles at oil/water interfaces (San-Miguel and Behrens, 2012; Zanini et al., 2017). The roughness of surface enlarges the surface area of the colloidal particles and consequently increases the adsorption energy of particles at oil/water interface, which is shown as follows:

$$\begin{array}{r} \Delta F\;=\;-\pi r^{2}\gamma_{O/W}{\left(1\;-\;\alpha |cos\theta |\right)}^{2} \end{array}$$

where α is the surface area magnification factor, θ is the contact angle for the smooth particles with the same surface chemistry (Nonomura et al., 2006).

**Figure 11:**
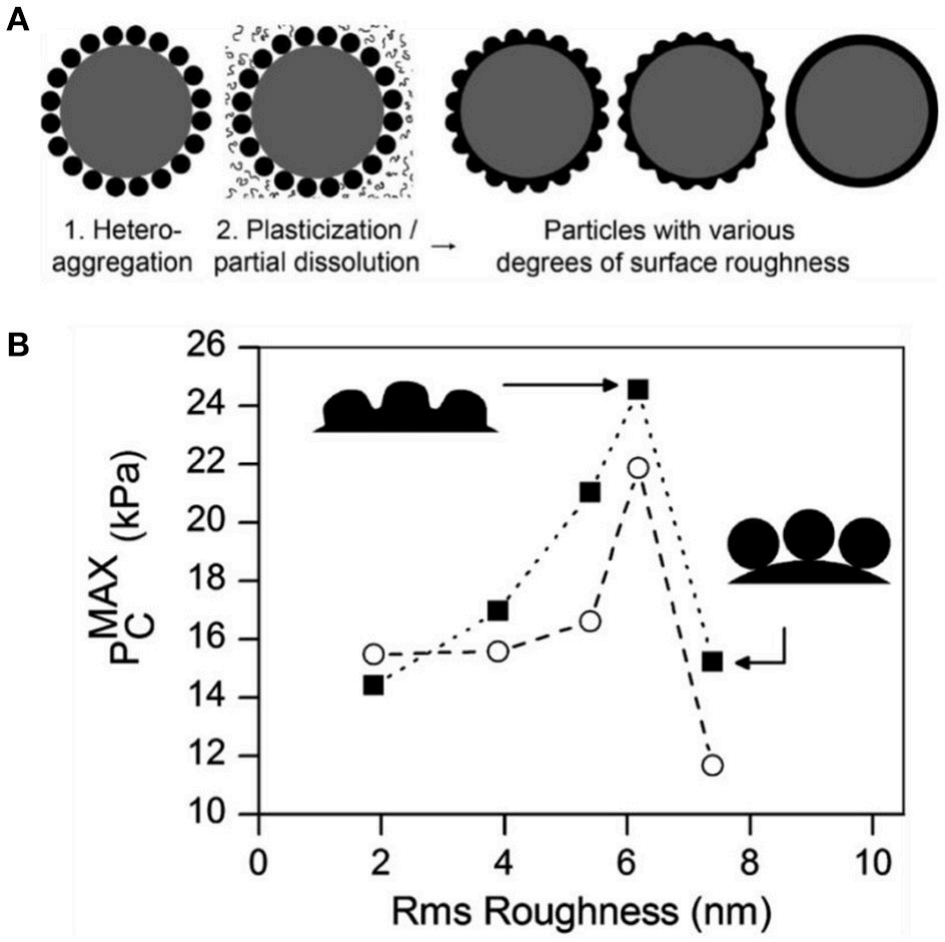
Schematic illustration of fabrication of particles with different surface roughness **(A)** and maximum capillary pressure for decane-in-water emulsions stabilized by particles with different surface roughness **(B)** (San-Miguel and Behrens, 2012). Reprinted with permission from San-Miguel and Behrens (2012). Copyright 2012 American Chemical Society.

San-Miguel and coworkers first reported the connection between particle roughness and the stability of related Pickering emulsions (San-Miguel and Behrens, 2012). Rough particles were fabricated by electrostatically adsorbing of negatively charged 50 nm poly(methacrylic acid-*co*-methyl methacrylate) nanoparticles onto amine modified silica particles as shown in Figure 11. The surface roughness was finely tuned by fusing the polymer nanoparticles through exposure to aqueous solutions of acetone or ethanol with different concentrations. The emulsion stability stabilized by the as-prepared rough particles was quantitatively measured by maximum capillary pressure (${\text{P}}_{\text{C}}^{\text{MAX}}$), which was determined by centrifugation. It was found that the emulsion stability was greatly increased with the surface roughness in the Wenzel regime (with roughness below 6 nm). As the roughness increased further, the wetting regime of particles inverted to Cassie-Baxter wetting and consequently the benefit of surface roughness for emulsion stability was lost.

Zanini and coworkers employed the similar protocol to fabricate rough particles through electrostatic-driven absorption of negatively charged silica nanoparticles onto positively charged silica microparticles (Zanini et al., 2017). By contrast, the surface roughness was tuned by growing silica layer on the surface of raspberry-like particles using a modified Stöber process. It was found that surface roughness provides plenty of pinning points for the three-phase contact line and the pinning induces contact angle hysteresis. Consequently, the inversion of particle wettability happens for sufficient roughness. In other words, the particles with specific roughness can stabilize both W/O and O/W emulsions and the emulsion type only depend on the initial location of the particles.

## Conclusions and prospect

The review describes the up-to-date examples of tailoring wettability of particulate emulsifiers for Pickering emulsions. There are many reports tuning the wettability by changing the surface chemistry with small molecules or polymers via either physical adsorption or chemical anchoring. The *in situ* modification with amphiphiles render particles tunable wettability depending on the interaction between particles and amphiphiles. Especially, there has been an increasing interest in switchable Pickering emulsions, which are triggered by wettability change depending on environmental stimuli. Recently, the surface roughness was found to be another main factor affecting the wettability of particles. In addition to tune the surface chemistry, precisely control of the surface roughness also adjust the wetting behavior of particles at oil/water interface and consequently affect emulsion characteristics of the resultant Pickering emulsions. We foresee that complexed Pickering emulsions including multiple emulsions, water-in-water (W/W) emulsions, and oil-in-oil (O/O) emulsions can be produced with finely tailoring both the surface chemistry and surface roughness. With the incorporation of functional and responsive building blocks to particulate emulsifiers, the as-prepared Pickering emulsions may find emerging applications in biphasic catalysis, oil recovery and biomedical applications (Pera-Titus et al., 2015; Tang et al., 2015; Wu and Ma, 2016; Binks, 2017).

## Author contributions

LH and MX searched the literatures for the article and produced the first draft. All the authors discussed the content and contributed to review and edit the manuscript.

### Conflict of interest statement

The authors declare that the research was conducted in the absence of any commercial or financial relationships that could be construed as a potential conflict of interest.
